# Functional and Structural Characterization of *Pediococcus pentosaceus*-Derived Biosurfactant and Its Biomedical Potential against Bacterial Adhesion, Quorum Sensing, and Biofilm Formation

**DOI:** 10.3390/antibiotics10111371

**Published:** 2021-11-09

**Authors:** Mohd Adnan, Arif Jamal Siddiqui, Walid Sabri Hamadou, Syed Amir Ashraf, Md Imtaiyaz Hassan, Mejdi Snoussi, Riadh Badraoui, Arshad Jamal, Fevzi Bardakci, Amir Mahgoub Awadelkareem, Manojkumar Sachidanandan, Mitesh Patel

**Affiliations:** 1 Department of Biology, College of Science, University of Hail, Hail P.O. Box 2440, Saudi Arabia; arifjamal13@gmail.com (A.J.S.); walidsabrimail@gmail.com (W.S.H.); snmejdi@yahoo.fr (M.S.); badraouir@yahoo.fr (R.B.); arshadjamalus@yahoo.com (A.J.); fevzi.bardakci@gmail.com (F.B.); 2 Department of Clinical Nutrition, College of Applied Medial Sciences, University of Hail, Hail P.O. Box 2440, Saudi Arabia; amirashrafy2007@gmail.com (S.A.A.); mahgoubamir22@gmail.com (A.M.A.); 3 Center for Interdisciplinary Research in Basic Sciences, Jamia Millia Islamia, Jamia Nagar, New Delhi 10025, India; mihassan@jmi.ac.in; 4 Laboratory of Genetics, Biodiversity and Valorisation of Bioresources, High Institute of Biotechnology, University of Monastir, Monastir 5000, Tunisia; 5 Section of Histology-Cytology, Medicine Faculty of Tunis, University of Tunis El Manar, La Rabta-Tunis 1007, Tunisia; 6 Department of Oral Radiology, College of Dentistry, University of Hail, Hail P.O. Box 2440, Saudi Arabia; smanojk68@gmail.com; 7 Bapalal Vaidya Botanical Research Center, Department of Biosciences, Veer Narmad South Gujarat University, Surat 395007, India

**Keywords:** biosurfactants, *Pediococcus pentosaceus*, antibiofilm, anti-adhesion, antimicrobial, probiotic, lactic acid bacteria, quorum sensing

## Abstract

Biosurfactants are surface-active molecules of microbial origin and alternatives to synthetic surfactants with various applications. Due to their environmental-friendliness, biocompatibility, biodegradability, effectiveness to work under various environmental conditions, and non-toxic nature, they have been recently recognized as potential agents with therapeutic and commercial importance. The biosurfactant produced by various probiotic lactic acid bacteria (LAB) has enormous applications in different fields. Thus, in vitro assessment of biofilm development prevention or disruption by natural biosurfactants derived from probiotic LAB is a plausible approach that can lead to the discovery of novel antimicrobials. Primarily, this study aims to isolate, screen, and characterize the functional and biomedical potential of biosurfactant synthesized by probiotic LAB *Pediococcus pentosaceus* (*P. pentosaceus*). Characterization consists of the assessment of critical micelle concentration (CMC), reduction in surface tension, and emulsification index (% EI24). Evaluation of antibacterial, antibiofilm, anti-QS, and anti-adhesive activities of cell-bound biosurfactants were carried out against different human pathogenic bacteria (*B. subtilis*, *P. aeruginosa*, *S. aureus*, and *E. coli*). Moreover, bacterial cell damage, viability of cells within the biofilm, and exopolysaccharide (EPS) production were also evaluated. As a result, *P. pentosaceus* was found to produce 4.75 ± 0.17 g/L biosurfactant, which displayed a CMC of 2.4 ± 0.68 g/L and reduced the surface tension from 71.11 ± 1.12 mN/m to 38.18 ± 0.58 mN/m. *P. pentosaceus* cells bound to the crude biosurfactant were found to be effective against all tested bacterial pathogens. It exhibited an anti-adhesion ability and impeded the architecture of the biofilm matrix by affecting the viability and integrity of bacterial cells within biofilms and reducing the total EPS content. Furthermore, the crude biosurfactant derived from *P. pentosaceus* was structurally characterized as a lipoprotein by GC-MS analysis, which confirms the presence of lipids and proteins. Thus, our findings represent the potent anti-adhesion and antibiofilm potential of *P. pentosaceus* crude biosurfactant for the first time, which may be explored further as an alternative to antibiotics or chemically synthesized toxic antibiofilm agents.

## 1. Introduction

Adherence of bacteria on living and non-living surfaces is the first and most important step in the formation of biofilms, a natural phenomenon of most bacteria producing extracellular polymeric matrix in response to various environmental cues. Presence of biofilm is everywhere in nature, which has a severe impact, and ultimately huge losses to the food, dairy, oceanic, aquaculture, beverage, environment, and biomedical industries [1,2]. Various evidence suggests that microbial life in biofilm mode leads to increased resistance toward host defense systems, antibiotics, and other modes of actions [3,4,5,6,7,8,9]. On that account, bacterial species can grow resistant to disinfectants, which creates many hurdles in removing the biofilm. Therefore, biofilm removal is a global challenge that necessitates developing novel natural bioactive compounds to control biofilms as an alternative to antibiotics or chemically synthesized antibiofilm agents.

Recently, microbial surfactants, commonly known as ‘biosurfactants’, have caught the attention of scientists due to their environmentally-friendly nature, easy bulk production, effectiveness under harsh environmental conditions, selectivity, diversity, and potential applications in different fields. Biosurfactants are amphiphilic substances commonly synthesized by different microbes on their surface or as extracellular secretion, which efficiently reduces the surface tension and represent emulsifying activities [10]. Biosurfactants are surface-active compounds that consist of protein–polysaccharide complexes, glycolipids, phospholipids, neutral lipids, fatty acids, rhamnolipids, and lipopeptides [11]. Various biosurfactants have displayed antimicrobial and anti-adhesive properties, leading them as suitable candidates to combat a variety of infections and biofilms caused by diverse microorganisms [12].

Being an essential part of natural microbiota, probiotic LABs are known to have some potent biological properties, as they can produce many antimicrobial agents including biosurfactants [8]. Plenty of studies have been documented and show the potentiality of probiotic LAB as producers of biosurfactants. Furthermore, these studies also document the applications of isolated biosurfactants from LAB in impeding the adhesion of microbes, desorption activity, and inhibiting the development of biofilm on a variety of surfaces such as silicone, rubber, polypropylene, and different biomedical instruments/implants [13,14,15,16,17,18,19,20,21,22]. One study tested 15 *Lactobacillus* strains in vitro for biosurfactant production, and all tested strains were able to produce biosurfactants during their mid-exponential and stationary growth phases [23]. Studies in the past have demonstrated the ability of a probiotic bacteria-derived biosurfactant to inhibit the adhesion in the case of *Enterococcus faecalis* to a glass surface and staphylococcal biofilm formation, respectively. This shows the biosurfactant’s valuable attributes and their possible use as an anti-adhesive and antibiofilm agent [24,25]. Biosurfactants are believed to interfere in microbial adhesion and desorption processes via altering the hydrophobicity of the surface [26,27]. As a result, prior treatment of medical implants with potential biosurfactants can be used as a targeted and preventive strategy against biofilm formation by multi-drug resistance bacteria on the wound site, medical insertion materials as well as inert surfaces in a hospital environment [24,25,28,29,30,31].

Various biosurfactants have displayed powerful antimicrobial and anti-adhesive properties, making them a suitable candidate to combat biofilms and related infections caused by diverse microorganisms. As per our knowledge, there are no reports on the potential of *P. pentosaceus* biosurfactants as an antibiofilm agent, which can be used for various biomedical and industrial applications. Therefore, the present study is divided into two goals. First, to isolate, screen, and characterize the functional ability of biosurfactants synthesized by probiotic LAB *P. pentosaceus* isolated from the curd sample. The biosurfactant production screening of *P. pentosaceus* was tested via different qualitative and quantitative methods such as drop collapse, oil displacement, C-TAB agar plate, and emulsification method, followed by gas chromatography-mass spectrometry (GC-MS) analysis. Second, the biomedical potential (antibacterial, antibiofilm, and anti-adhesive) assessment of cell-bound *P. pentosaceus* biosurfactants against various human pathogenic bacteria.

## 2. Results

### 2.1. Identification and Screening of Promising Biosurfactant Producing Lactic Acid Bacteria

**Figure 1:**
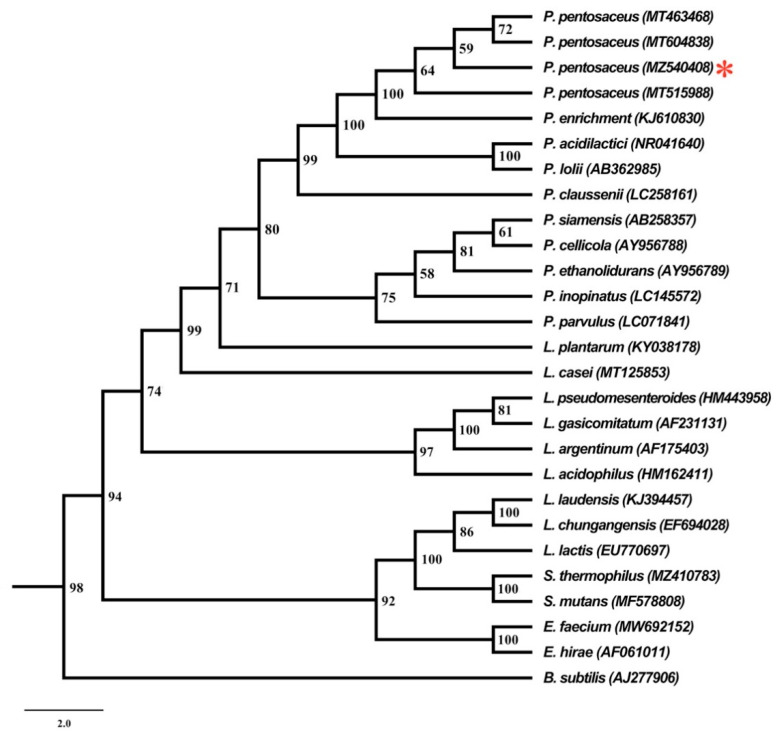
Phylogenetic tree representing the evolutionary relationship of *P. pentosaceus* with other strains from the NCBI database. * denotes our isolated strain *P. pentosaceus*.

Based on morphological and 16S rRNA sequence analysis, isolated probiotic LAB was identified as *P. pentosaceus.* The GenBank sequence database was used after the BLASTn homology run of the obtained nucleotide sequence of the strain MBP003. More than 99% of sequence identity was matched with *P. pentosaceus* against the nucleotide sequence collection in the database. Following successful identification, the nucleotide sequence with accession number MZ540408 was deposited to the GenBank database of NCBI. The evolutionary relationship of *P. pentosaceus* is represented in Figure 1.

Biosurfactant producing capability of isolated *P. pentosaceus* cell-free solution was screened via different qualitative and quantitative assays (Supplementary Figure S1). First, a drop collapse assay was performed, which is based on the droplet destabilization by the surfactants. Accordingly, a drop of a cell-free biosurfactant solution of *P. pentosaceus* was dispensed on oil. On this occasion, if the surfactant is not present in the liquid, the drop is stable, meaning that from hydrophobic sites, the polar water molecules are repulsed. In contrast, if the surfactant is present in the liquid, the drop will collapse. This is due to the interfacial tension or force between the hydrophobic surface and liquid. In the case of *P. pentosaceus*, the flattened drop of the supernatant placed over the surface of oil suggested the existence of the biosurfactant. Additionally, the oil spreading assay was also performed as a confirmatory assay for the validation of the drop collapse assay result. In this assay, the area of oil displacement is directly proportional to the concentration of the surfactants. In the case of *P. pentosaceus*, the oil spread assay was conducted in relation to the diameter and time in which the *P. pentosaceus* cell-free biosurfactant solution revealed positive results (Table 1).

The emulsification assay is a quantitative method that is indirectly used for the screening of biosurfactant production. It is assumed that when a biosurfactant is present in the cell-free culture, it would emulsify the hydrocarbon substrate. In the present study, the emulsification assay of the *P. pentosaceus* cell-free biosurfactant solution was carried out against n-hexadecane. Normally, an emulsification index of 30% is regarded as positive activity [8]. In the case of *P. pentosaceus*, the cell-free biosurfactant solution showed a 39.73 ± 1.59% emulsification index against n-hexadecane.

The BAP assay is another semi-quantitative method for the confirmation of biosurfactant production. When the cell-free biosurfactant solution of *P. pentosaceus* was added into the wells on BAP plates, dark blue halos that resulted around the wells confirmed the presence of biosurfactants (Table 1).

### 2.2. Growth Kinetics and Biosurfactant Production

**Figure 2:**
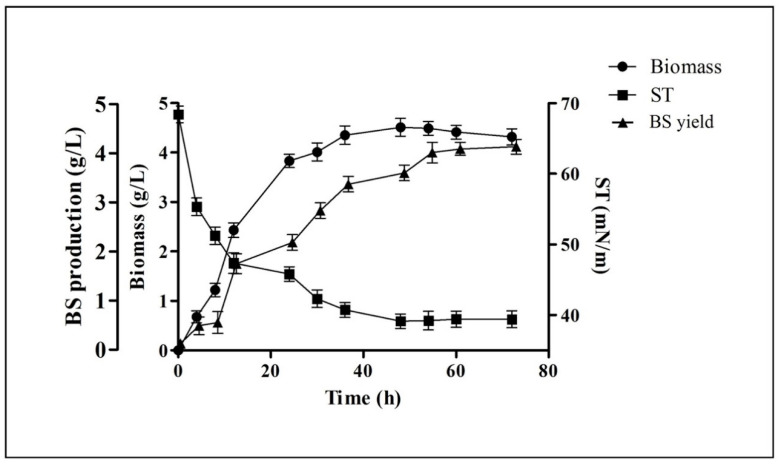
Profiling of growth kinetics of *P. pentosaceus* in reference to the reduction in surface tension (ST) and production the biosurfactant (BS) at different time intervals. Values are represented as mean ± SD of three independent experiments.

Biosurfactant production and extraction were carried out in MRS-Lac medium. Figure 2 represents the kinetic profile plot of biosurfactant production by *P. pentosaceus* in this medium. It shows that the synthesis of the biosurfactant was growth-dependent and took place in the log phase. A graph of surface tension reduction was plotted. Moreover, cell biomass (4.51 ± 0.18 g/L), biosurfactant production (4.75 ± 0.17 g/L), and the highest reduction activity (39.12 ± 1.03 mN/m) were found/noted when the cells entered in their stationary growth phase. Production of a biosurfactant and reduction in the surface tension was constant up to the termination point of the stationary growth phase.

### 2.3. Physical Properties of the Biosurfactant

**Figure 3:**
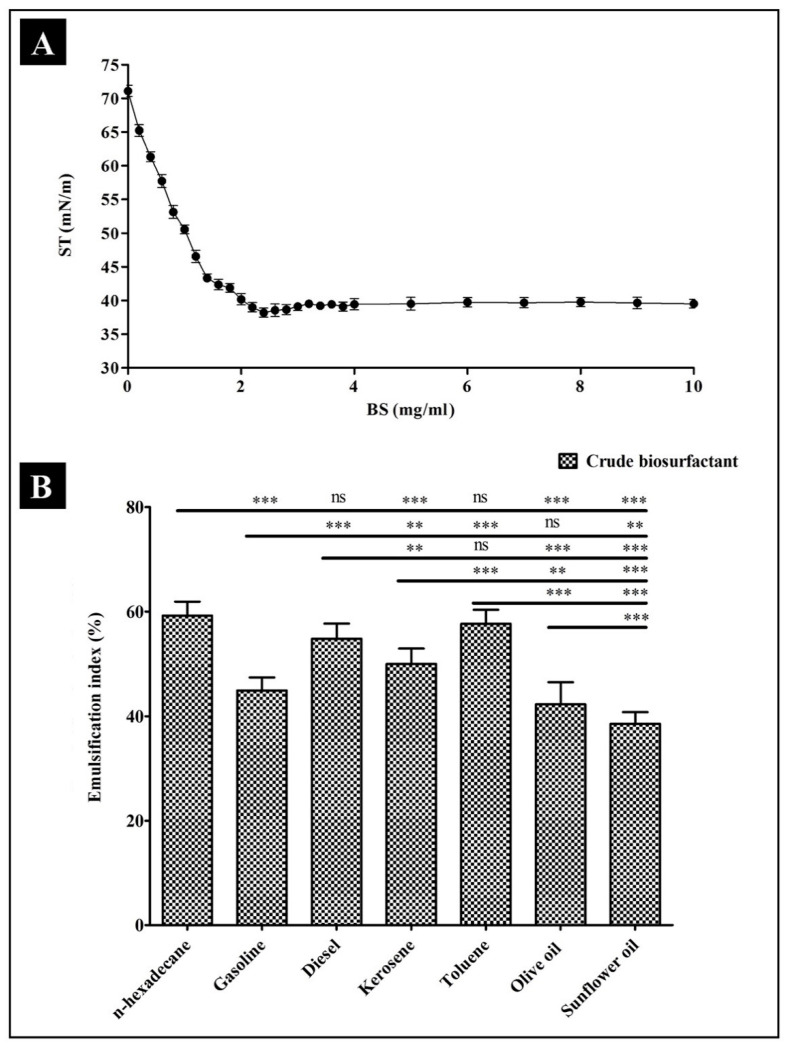
Results of the physical properties of *P. pentosaceus* crude biosurfactant (BS). (**A**) Progressive decrease in surface tension (ST) with an increase in the concentration of the biosurfactant up to 2.4 g/L. (**B**) %E24 of the cell-bound biosurfactant against different substrates. Values are represented as mean ± SD of three independent experiments. Significance; ns > 0.05, * *p* < 0.05, ** *p* < 0.005, *** *p* < 0.0005.

Decreasing the surface tension at the lowest CMC is one of the crucial attributes of an efficient biosurfactant. The extracted cell-bound biosurfactant was further evaluated for a reduction in surface tension and CMC value by using a tensiometer (K11, Kruss, Hamburg, Germany). A sudden break in the surface tension plot versus the plot of biosurfactant concentration is defined as the CMC. Biosurfactant produced by *P. pentosaceus* reduced the surface tension from 71.11 ± 1.12 mN/m to 38.18 ± 0.58 mN/m at a CMC of 2.4 ± 0.68 g/L (Figure 3A). Moreover, the biosurfactant produced by *P. pentosaceus* was also able to emulsify different hydrocarbon substrates such as n-hexadecane, gasoline, diesel, kerosene, toluene, olive oil, and sunflower oil. The highest %EI24 was obtained for the n-hexadecane/biosurfactant emulsion (59.22 ± 1.55), while the lowest %EI24 was obtained for the sunflower oil/biosurfactant emulsion (38.55 ± 1.28) (Figure 3B).

### 2.4. Antibacterial Activity of P. pentosaceus Crude Biosurfactant

**Figure 4:**
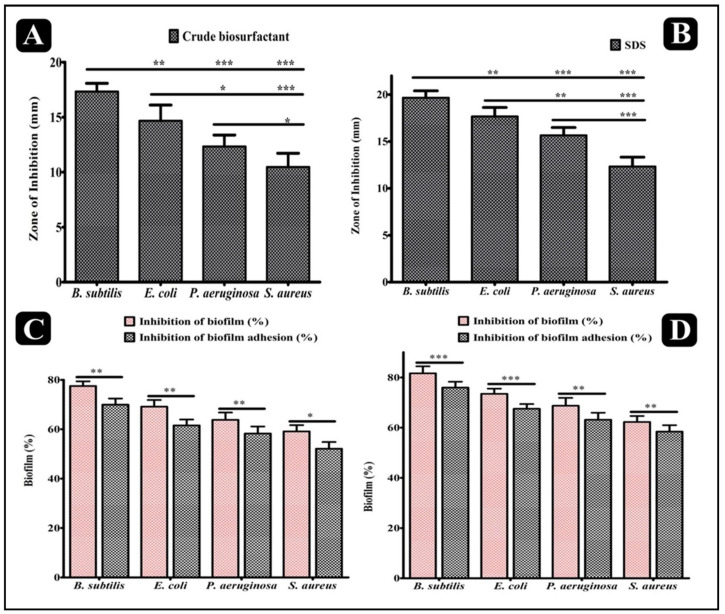
Results of the antibacterial and antibiofilm activity of *P. pentosaceus* crude biosurfactant and standard SDS. (**A**) Antibacterial activity of *P. pentosaceus* crude biosurfactant (100 mg/mL) against different bacterial pathogens. (**B**) Antibacterial activity of standard SDS surfactant (1% *v*/*v*) against different bacterial pathogens. (**C**) Effect of *P. pentosaceus* crude biosurfactant on established biofilms and on adherence ability of different bacterial pathogens at respective MICs. (**D**) Effect of the standard SDS surfactant on the established biofilms and on the adherence ability of different bacterial pathogens at respective MICs. Values are represented as the mean ± SD of three independent experiments. Significance; ns > 0.05, * *p* < 0.05, ** *p* < 0.005, *** *p* < 0.0005.

The inhibitory potential of crude biosurfactant of *P. pentosaceus* and standard SDS (sodium dodecyl sulfate) was determined via the agar cup/well diffusion method against four different biofilm-forming human pathogens: Gram-negative (*E. coli* and *P. aeruginosa*) and Gram-positive (*B. subtilis* and *S. aureus*). Both the *P. pentosaceus* crude biosurfactant and standard SDS displayed considerable an antibacterial effect against all the tested bacterial pathogens, represented in the form of the zone of inhibition (Figure 4A,B). The antibacterial potency of *P. pentosaceus* crude biosurfactant and standard SDS was further evaluated by assessing the minimum inhibitory concentration (MIC) and minimum bactericidal concentration (MBC) against the test pathogens. The values of *P. pentosaceus* crude biosurfactant MIC were ranged from 6.25–25 mg/mL and MBC values were found two-times higher than the MIC values (Table 2). The values of standard SDS MIC ranged from 0.2–0.8% and MBC values were 0.4 to 1.0% (Table 2).

### 2.5. Antibiofilm Potential of P. pentosaceus Crude Biosurfactant

The antibiofilm potential of the *P. pentosaceus* crude biosurfactant and standard SDS was determined by its capability to impair the preformed biofilms of the test strains and inhibit their adhesion ability to the surface. Our results showed that the *P. pentosaceus* crude biosurfactant and standard SDS efficiently disrupted the preformed biofilms with an ability to inhibit the adhesion potential of all test strains at MIC. At this concentration, the eradication of preformed biofilms by the *P. pentosaceus* crude biosurfactant was about 77.47 ± 1.11% for *B. subtilis*, 69.12 ± 1.56% for *E. coli,* 63.81 ± 1.70% for *P. aeruginosa*, and 59.10 ± 1.46% for *S. aureus*, respectively (vs. 81.65 ± 1.61% for *B. subtilis*, 73.49 ± 1.19% for *E. coli,* 68.72 ± 1.80% for *P. aeruginosa*, and 62.28 ± 1.36% for *S. aureus*, respectively, for standard SDS). The adhesion potential of biofilms was also found to decrease with percentage of eradication as 69.93 ± 1.44% for *B. subtilis*, 61.54 ± 1.38% for *E. coli*, 58.26 ± 1.64% for *P. aeruginosa*, and 52.13 ± 1.55% for *S. aureus*, respectively (vs. 75.98 ± 1.34% for *B. subtilis*, 67.53 ± 1.08% for *E. coli,* 63.17 ± 1.60% for *P. aeruginosa*, and 58.43 ± 1.47% for *S. aureus*, respectively, for standard SDS) (Figure 4C,D).

### 2.6. Effect of P. pentosaceus Crude Biosurfactant on Bacterial Cells Entrapped in Biofilms

**Figure 5:**
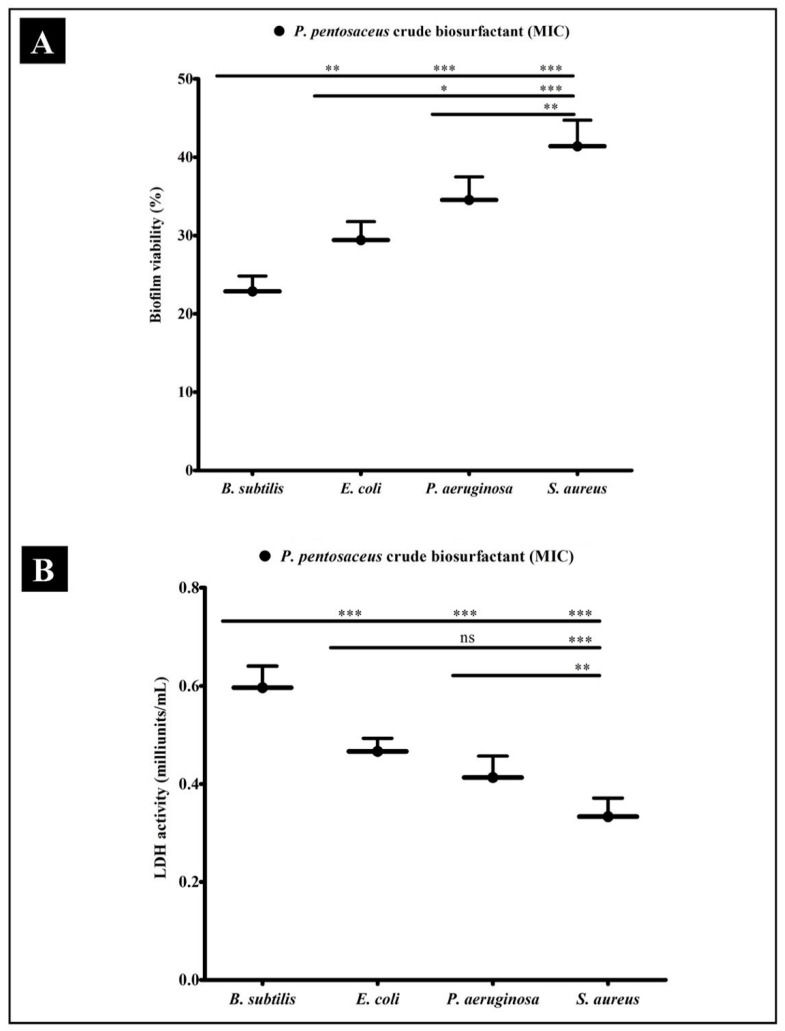
Results of the XTT and LDH assays for confirming the antibiofilm potential of biosurfactant. (**A**) Percentage of bacterial viability within biofilms determined by XTT assay at respective MICs. (**B**) Bacterial cell damage within the biofilm, based on LDH activity upon the treatment of *P. pentosaceus* crude biosurfactant at respective MICs. Values are represented as mean ± SD of three independent experiments. Significance; ns > 0.05, * *p* < 0.05, ** *p* < 0.005, *** *p* < 0.0005.

To determine the prospect that the *P. pentosaceus* crude biosurfactant could decrease the viability of bacteria within the biofilms, XTT (2,3-Bis(2 methoxy-4-nitro-5-sulfophenyl)-5-[(phenyl-amino)carbonyl]-2H-tetrazoliumhydroxide) reduction assay and LDH (lactate dehydrogenase) activity was performed. Obtained results revealed that viability of all bacteria inside biofilms were remarkably reduced upon treatment of *P. pentosaceus* crude biosurfactant with different susceptivity (Figure 5A). Similarly, LDH activity was also assessed. LDH is the bacterial intrinsic intracellular enzyme, which carries out the conversion of lactate into pyruvate and the reverse. LDH activity is well detected when the bacterial cell membrane is not intact. Our results indicated that, upon the treatment of the *P. pentosaceus* crude biosurfactant at the MIC level, the activity of LDH was found to be elevated in the supernatant of all test strains. Higher LDH activity was found in *B. subtilis,* whereas lower activity was found in *S. aureus* (Figure 5B). Hence, such results evidently indicate that the *P. pentosaceus* biosurfactant could impair the bacterial cell membrane within the biofilms and eventually bacterial death.

### 2.7. Effect of P. pentosaceus Crude Biosurfactant on Exopolysaccharide (EPS) Production

**Figure 6:**
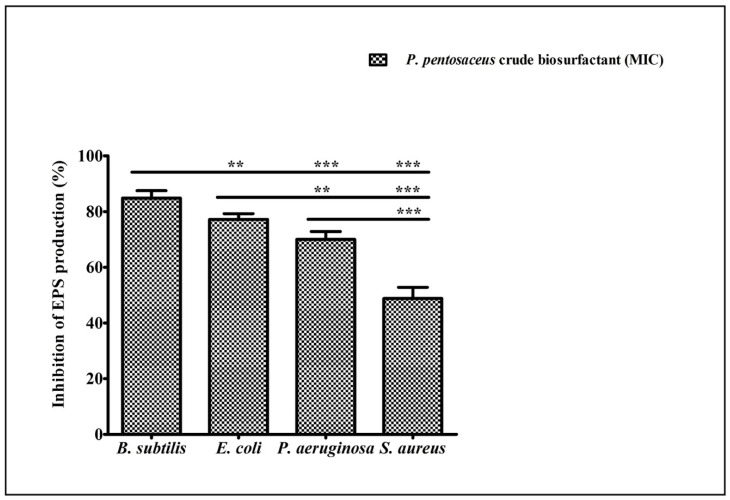
Inhibition of total EPS production by test pathogens in the presence of the *P. pentosaceus* crude biosurfactant at their respective MICs. Values are represented as mean ± SD of three independent experiments. Significance; ns > 0.05, * *p* < 0.05, ** *p* < 0.005, *** *p* < 0.0005.

EPS are biopolymers of bacterial origin and immersed within the biofilm. Biopolymers of EPS develop a matrix and are hydrated by retaining the water and keeps the cells together within the biofilm. Our results showed that the production of total EPS was considerably decreased in all test strains after the treatment of the *P. pentosaceus* crude biosurfactant (Figure 6).

### 2.8. Microscopic Analysis for the Visualization of Disrupted Biofilms by Light (LM) and Scanning Electron (SEM) Microscopy

**Figure 7:**
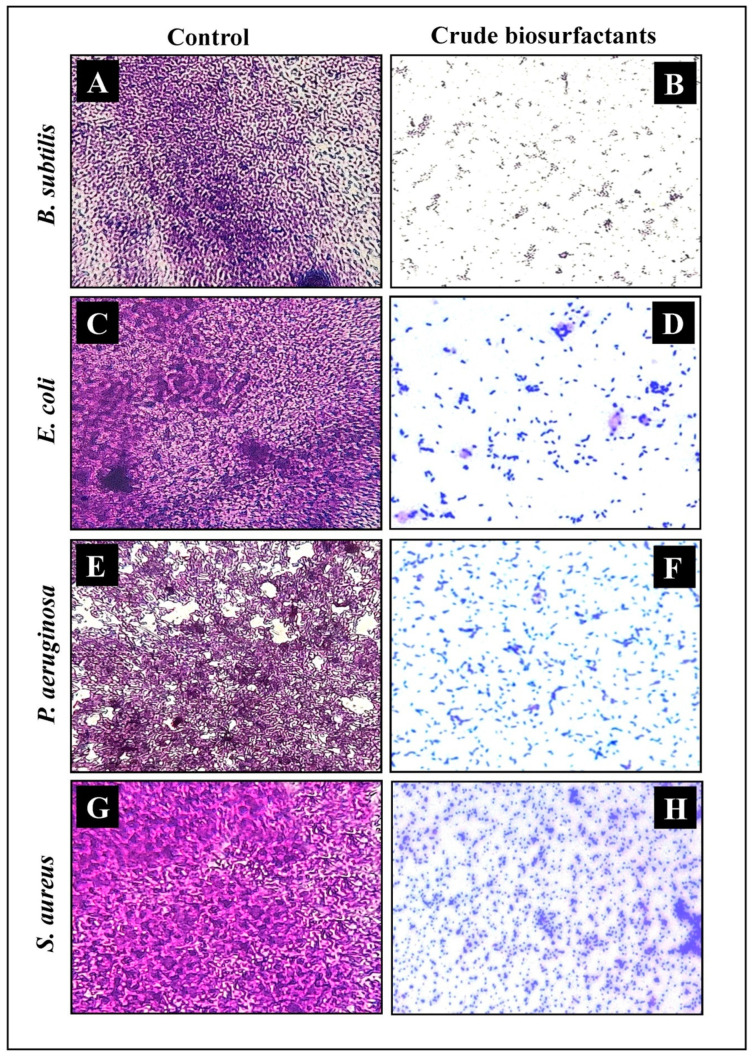
Micrographs of disrupted matured biofilms of the test pathogens by *P. pentosaceus* crude biosurfactant formed on the glass surface at their respective MICs under LM. Growth control (**A**,**C**,**E**,**G**), with the *P. pentosaceus* crude biosurfactant (**B**,**D**,**F**,**H**).

**Figure 8:**
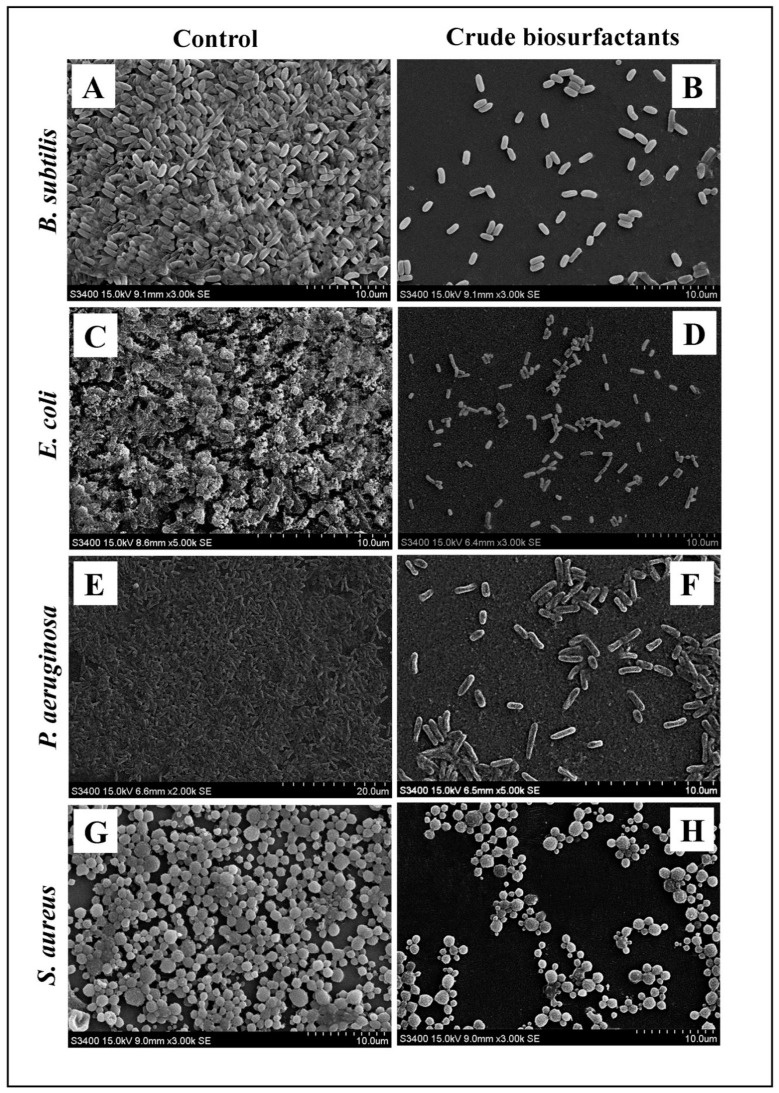
Micrographs of the disrupted matured biofilms of the test pathogens by the *P. pentosaceus* crude biosurfactant formed on a glass surface at their respective MICs under SEM. Growth control (**A**,**C**,**E**,**G**), with *P. pentosaceus* crude biosurfactant (**B**,**D**,**F**,**H**).

The efficiency and level of biofilm disruption of test strains by the *P. pentosaceus* crude biosurfactant and its MIC were investigated under LM and SEM. In light microscopy, the control sample displayed a heavy-knit like mat of biofilms, while in the presence of the *P. pentosaceus* crude biosurfactant, the deterioration in biofilm thickness with a lower appearance of microcolonies was observed (Figure 7). Similarly, biofilm anatomy and surface morphology were confirmed by SEM analysis in the presence and absence of the *P. pentosaceus* crude biosurfactant. In the control group of samples, multi-tiered biofilm growth was seen, while the treatment group with the *P. pentosaceus* crude biosurfactant displayed a reduction in the thick aggregation of the test bacterial cells (Figure 8). This might be due to the impairment of the EPS layer present in the biofilms. These results were further confirmed by the performed EPS assay. Our results of the EPS assay displayed a remarkable reduction in the EPS production of all test strains treated with the *P. pentosaceus* crude biosurfactant. Altogether, our results have evidently demonstrated the effectiveness of the *P. pentosaceus* crude biosurfactant as potential, natural, and green antibiofilm agent.

### 2.9. Anti-Quorum Sensing (QS) Activity

The *P. pentosaceus* crude biosurfactant was tested for QS modulatory activity against *C. violaceum*. The crude biosurfactant showed significant inhibition of pigment; a determinant of anti-QS activity as well as the inhibition of *C. violaceum* growth. The crude biosurfactant demonstrated interferences in QS activity as shown by reduced violacein production by *C. violaceum*. The crude biosurfactant inhibited the violacein production up to 87.39% at the concentration of 100 mg/mL.

### 2.10. GC-MS Analysis

**Figure 9:**
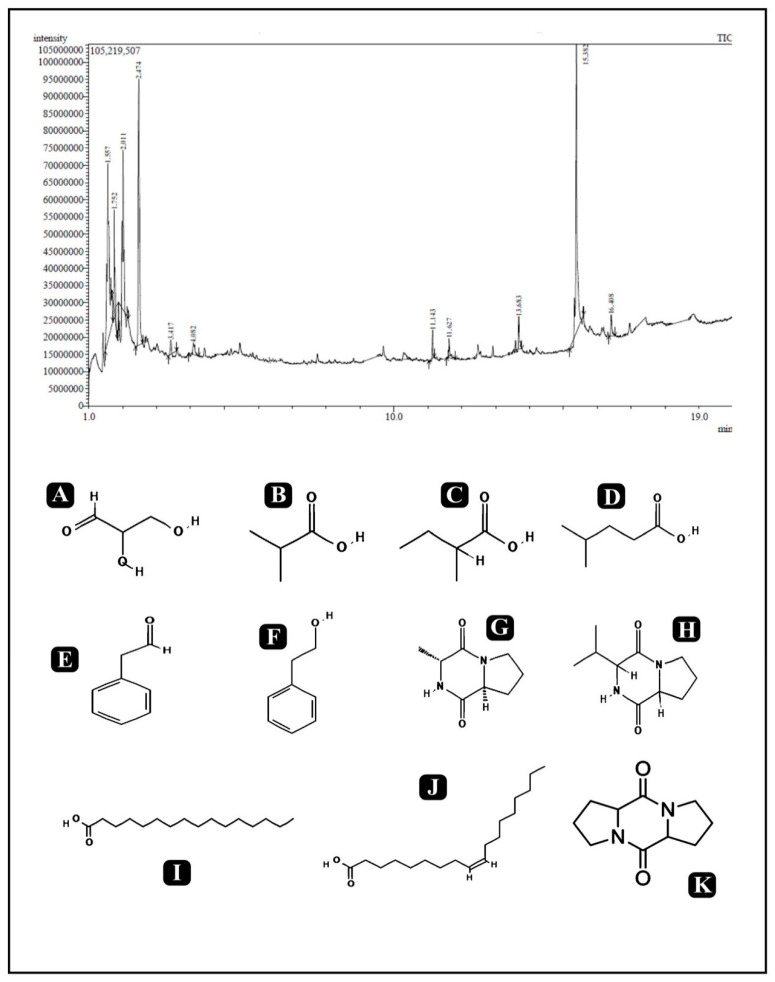
GC-MS analysis of crude biosurfactant derived from *P. pentosaceus.* Identified compounds. (**A**) Glyceraldehyde, (**B**) Propanoic acid, 2-methyl-, (**C**) Butanoic acid, 2-methyl-, (**D**) Pentanoic acid, 4-methyl-, (**E**) Benzeneacetaldehyde, (**F**) Phenylethyl Alcohol, (**G**) (Cyclo(-d-Ala-L-Pro)), (**H**) (Cyclo(-Pro-Val)), (**I**) n-Hexadecanoic acid, (**J**) Oleic Acid, and (**K**) Octahydro-5H, 10H-dipyrrolo [1,2-a:1′,2′-d]pyrazine-5,10-dione.

The crude biosurfactant derived from *P. pentosaceus* was analyzed by GC-MS for the determination of compounds present in it. Results represent the different major peaks of the corresponding fatty acids, fatty acid esters, sugars, and dipeptides as listed in Table 3 and the obtained chromatogram with a chemical structure of identified compounds is shown in Figure 9.

## 3. Discussion

Biofilm formation is a major concern around the world due to the bacterial ability to adhere and grow as biofilms on any type of surface. Within the biofilm, bacterial cells are resistant to antimicrobial agents and other environmental or chemical fluctuations when compared to their planktonic form [32,33]. Such resistance toward the antimicrobial agent is critical and a matter of concern for many industries such as medical instrumentation, food, dairy, brewery, drinks and juices, aquaculture, marine, oceanic, etc. [2,34]. Moreover, bacterial biofilms also impact human lives by extensive means, as more than 60% of all bacterial infections are associated with the biofilm of bacteria developed on different medical devices/implants. Therefore, there is an immediate need to consider the biofilms as a target for pharmacological development, and novel approaches are needed to control or inhibit the biofilm formation [1].

Biosurfactants have recently received the considerable attention of researchers mainly due to their high selectivity, eco-friendly nature, and specific mechanism of action under harsh conditions such as pH, salinity, and temperature [35]. Biosurfactants have an extensive application in the food, oil, biodegradation, cosmetic, agriculture, pesticide, and medicine/pharmaceutical industries [36,37]. Biosurfactants have also proven their effectiveness in fighting against pathogenic bacteria and their biofilms [12]. Thus, in the search of a novel method/molecule for biofilm eradication, we have screened the biosurfactants produced by probiotic LAB *P. pentosaceus*. There are several strains of LAB reported for their antimicrobial activity, and inhibiting the formation of biofilms of many pathogenic microbes [19,24,38,39,40]. Therefore, in the present study, we exemplified and provided evidence with regard to the use of *P. pentosaceus* biosurfactants as a green alternative to antibiotics and chemically synthesized agents to inhibit the biofilm formation by pathogenic bacteria.

First, *P. pentosaceus* was screened for the production of biosurfactants via different qualitative and quantitative assays. Drop collapse, oil displacement, C-TAB agar plate, and emulsification assays are the most effectual tools to confirm the production of biosurfactants and to identify distinct types of biosurfactants in bacterial strains [41]. Microbial biosurfactants have many primacies than chemical surfactants such as biodegradability, low toxicity, and ecological appropriateness, which is hugely beneficial in various applications. The production of biosurfactants was started in the log phase and was found to be growth-dependent. The highest surface tension reduction (39.12 ± 1.03 mN/m), cell biomass (4.51 ± 0.18 g/L), and biosurfactant production (4.75 ± 0.17 g/L) was found when the cells entered into their stationary growth phase. Production of biosurfactants and a reduction in the surface tension was found to be constant up to the end of the stationary growth phase (Figure 2). Other researchers have also reported a similarly notable reduction in surface tension while working with LAB. Rodrigues et al. (2004) found a significant reduction in surface tension from 72 to 39 mN/m and 72 to 37 mN/m while working with *Lactobacillus fermentum* RC-14 and *Streptococcus thermophilus* A, respectively [20].

Another remarkable characteristic of a biosurfactant is the generation of micelles, which are known as amphipathic molecule aggregates [42,43,44]. However, for application and use, it is essential to categorize efficient and effective biosurfactants. Measurement of effectiveness is carried out by the minimum value at which reduction in surface tension has occurred, whereas, the CMC of biosurfactant was carried out for the measurement of efficiency [45]. Reduction in surface tension starts once the concentration of the surfactant increases in the medium and the formation of micelles has occurred. CMC value of the biosurfactant produced by *P. pentosaceus* was found to be 2.4 ± 0.68 g/L when compared to the CMC value of the commonly used chemical surfactant SDS, which is 1.8 g/L. SDS is known to reduce surface tension from 72.0 to 37 mN/m [24,46,47]. Biosurfactants produced by *Lactobacillus delbrueckii* in peanut oil cake had CMC of 2 g/L [14]. The efficiency of any surfactant is assessed by its capability to lower the surface and interfacial tension of the production medium. One example of an efficient surfactant is one that can lower the surface tension of water from 72.0 to 35.0 mN/m [48]. Thus, our results of this study are in agreement with those derived for biosurfactants extracted from other LAB.

Currently, there is a high demand for natural and novel antimicrobial compounds due to the higher resistance of antimicrobial agents in clinical microbes, which has attracted the attention of many researchers to utilize biosurfactants as a new antimicrobial compound against such pathogens [10,23,49,50,51,52]. One of the possible modes of action behind the antimicrobial activity of a biosurfactant is that it can cause disruption of cytoplasmic membranes, which eventually leads to cell lysis, leakage of metabolites, and disrupting the confirmation of proteins, which in the end changes the important membrane functions [53,54]. The *P. pentosaceus* crude biosurfactant was found to be effectual against the Gram-negative and -positive bacteria at different degrees.

Determination of MIC and MBC are important and relatively economical methods to evaluate the potency of many antimicrobial agents. In this study, the MIC and MBC values of *P. pentosaceus* crude biosurfactants were about 6.25 and 12.5 mg/mL for *B. subtilis*, 12.5 and 25 mg/mL for *E. coli* and *P. aeruginosa*, and 25 and 50 mg/mL for *S. aureus*, respectively. The antimicrobial activity of the biosurfactants isolated from a diverse genera of microorganisms have been reported [55,56]. However, the antimicrobial properties of the crude biosurfactant obtained from *P. pentosaceus* against pathogenic bacteria were found equivalent to that obtained with the crude biosurfactants produced by several other species of LAB such as *L. jensenii*, *L. rhamnosus, L. lactis* 53, *S. thermophilus* A, and *L. helveticus* MRTL91, among various MIC concentrations between 25 and 100 mg/mL. Biosurfactant produced by *S. thermophilus* A reported for antimicrobial properties against *C. tropicalis* at even very low biosurfactant MIC concentration of 2.5 mg/mL. Biosurfactants of *L. paracasei* were also reported for antimicrobial activity against *S. aureus, S. pyogenes*, and *S. agalactiae* with a biosurfactant MIC concentration of 25 mg/mL. In another study, antimicrobial activities of the biosurfactant produced by the *L. paracasei* ssp. *Paracasei* A20 showed maximal growth inhibition at a MIC concentration ranging between 25 and 50 mg/mL [8,19,21]. Overall, these results from previous studies are in line with our results, except for biosurfactants produced by *S. thermophilus* A against *C. tropicalis.* Moreover, results from pure biosurfactants such as lipopeptide isolated from the cell cultures of *L. acidophilus* and *L. pentosus* displayed potential bioactivities against several antagonists such as *Proteus mirabilis*, *S. aureus*, *Streptococcus pneumoniae*, *K. pneumoniae*, and *C. albicans* [57]. The MIC values revealed that the lipopeptide fraction had a stronger antimicrobial effect at lower MIC values ranging from 7.81 µg/mL (*P. mirabilis*) to 62.5 µg/mL (*K. pneumoniae*).

Furthermore, the anti-adhesive activity against all pathogens showed inhibition percentages ranging from 65% against *P. mirabilis* (250 µg/mL) to 93% against *K. pneumoniae* (250 µg/mL). In the same study, the efficacy of biosurfactants showed the maximum antibiofilm percentage against the tested strains [57]. Similarly, another study reported *Pseudomonas* strain UCMA 17988, isolated from raw cow milk, for its ability to produce a lipopeptide biosurfactant. The antimicrobial activity of the major isolated isoforms of the biosurfactant was observed against *S. aureus* with a MIC of 0.5 µg/mL against *L. monocytogenes* and *Salmonella enterica* with a MIC of 1 µg/mL [58].

The biosurfactant develops a film that alters the wettability of the native surface and alters the adhesion ability of pathogens [51]. There is one assumption that biosurfactants manipulate the bacterial–surface interface [59]. In this study, apart from antibacterial activity, *P. pentosaceus* crude biosurfactants also revealed notable results in inhibiting the biofilms in a concentration-dependent manner at their respective MICs against tested pathogenic bacterial strains. *P. pentosaceus* crude biosurfactants were found to be efficiently hampering the adhesion ability as well as impeding the preformed biofilms of the test strains (Figure 4B). Results of the XTT assay confirmed that viability of bacterial cells inside the biofilm was also affected by the crude biosurfactant from *P. pentosaceus* (Figure 5A). This means that *P. pentosaceus* crude biosurfactants could also affect the integrity of bacterial cells inside the biofilm. Moreover, with *P. pentosaceus* crude biosurfactant treatment, impaired cells possibly release the intrinsic intracellular enzyme LDH (Figure 5B).

Biofilm biomass was assessed using the gold standard method of the crystal violet assay. Our results showed that the *P. pentosaceus* crude biosurfactant was potentially efficient in inhibiting the biofilms of all test strains (Figure 6). This result was further confirmed by visualizing under SEM (Figure 7). Disrupted integrity of the cell walls and reduction in thickness of multi-layered biofilm growth can be seen. Furthermore, in the presence of the *P. pentosaceus* biosurfactant, all bacterial strains failed to develop as clusters as well as unable to maintain their typical morphology. This is due to the compromised cell walls. Another important aspect studied was EPS, which is produced by the bacteria and is vital for not only maintaining the structural integrity, but also substantially contributes in adhesion to various surfaces and microcolony development, leading to the formation of biofilms [60]. Our study displayed remarkable results in inhibiting the EPSs of all test strains by the *P. pentosaceus* crude biosurfactant. An EPS rich matrix is very crucial for maintaining the physical stability and attachment of biofilms [61]. Therefore, targeting the biochemical constitution of EPS ultimately destabilizes the biofilm matrix and its complexity, which further eases the drug access directly into the biofilms [62].

Antibiofilm potency of the biosurfactant derived from LAB has been reported against different pathogenic microbes [15,19,54,59,63,64,65]. Similarly, functions of biosurfactants from different LAB against the adhesion of microbes have been widely assessed and might be an important approach in direction to inhibit the adhesion and combating the colonization of pathogenic microbes on different type of surfaces [64,66]. Biosurfactants of *L. helveticus* MRTL91 exhibited lower anti-adhesive activity against *S. typhi*, *E. coli*, *P. aeruginosa*, *S. flexneri*, and *C. albicans* at same concentration.

Falagas and Makris (2009) proposed the efficacy of biosurfactants extracted from the probiotic microorganism in hospitals to hamper the colonization of pathogens onto the medical equipment/implants to control the nosocomial infections [17]. Rodrigues et al. (2006) reported the use of biosurfactants derived from *S. thermophilus* A to inhibit the colonization of microbial pathogens on silicone rubber [19]. Gudina et al. (2010) reported the anti-adhesive potential of biosurfactants produced by LAB and among the highest anti-adhesive activity at a concentration of 25 mg/mL was found against *S. aureus*, *S. epidermidis*, and *S. agalactiae* [67]. Antibiofilm capability of biosurfactants obtained from *L. paracasei* and *L. paracasei* ssp. *Paracasei* A20 has been also reported against several human pathogens and yeast strains, and around 75% of inhibition was found against different pathogens. Fracchia et al. (2010) also reported the anti-adhesive potential of biosurfactants derived from *Lactobacillus* sp. and the results showed a reduction in the biofilm by 85% at a 312.5 µg/mL concentration [68]. Biosurfactants derived from *L. helveticus* MRTL 91 was reported to inhibit the biofilm completely from the silicone tube at a 25 mg/mL concentration [19]. *L. acidophilus* derived biosurfactants are reported to inhibit more than 50% of the deposition of bacterial pathogens such as *E. coli, S. aureus*, *C. albicans*, *E. faecalis*, and *S. epidermidis*. The *L. fermentum* B54 derived biosurfactant also showed anti-adhesive activity against uropathogenic microorganisms [63,69]. A few more strains of LAB, which are able to produce biosurfactants, are also reported to decrease the biofilm formation of several pathogens [19,63,69]. Biosurfactants derived from *L. lactis* 53 also inhibited the growth of *R. cariosa* and *C. tropicalis* on silicone tubing.

Furthermore, in numerous bacteria, their virulence is controlled via QS (cell density dependent global gene control system). Hence, interfering with this system is manifested as a prospect approach to inhibit the pathogenicity of bacteria. For the screening of QS inhibitors, *C. violaceum* is used as the model organism because of the easy detection of the violacein pigment. Therefore, in this study, the *P. pentosaceus* crude biosurfactant was also tested for QS interference by inhibiting the violacein production by *C. violaceum*. The production of violet pigment is regulated by a CviIR-dependent QS system in *C. violaceum* [70]. Therefore, any modulation of this pigment in *C. violaceum* is regarded as the direct indication of QS intrusion. The same type of effect on the inhibition of violacein has been reported for many natural products [71,72]. Based on the inhibition of growth and violacein production in *C. violaceum*, the *P. pentosaceus* crude biosurfactant was found as an active QS inhibitor.

The GC-MS characterization of the crude biosurfactant was also carried out for the identification of characteristic compounds present in the sample. The fatty acid composition of the biosurfactant produced by *P. pentosaceus* showed the presence of n-hexadecanoic acid, oleic acid, propanoic acid, butanoic acid, and pentanoic acid. Apart from fatty acids, sugar (glyceraldehyde), alcohol (phenylethyl alcohol), aldehyde (Benzeneacetaldehyde), and dipeptides were also determined. Therefore, our results showed that the biosurfactant derived from *P. pentosaceus* is most likely a lipoprotein with a lipid and protein as a major part. This result of our findings is in accordance with the reported literature for lipoprotein from the *Pediococcus dextrinicus* SHU1593 strain [73]. The production of the lipoprotein type of biosurfactants from LAB has not yet been commonly reported, as the majority of the studies were reported on the production of glycolipids [7,26] or a mixture of proteins and polysaccharides associated with phosphate groups [74]. Therefore, it can be concluded that this strain of *P. pentosaceus* producing lipoprotein type biosurfactants has antibacterial and antibiofilm potential, which can be used for large scale biosurfactant production.

## 4. Materials and Methods

### 4.1. Isolation and Screening of Lactic Acid Bacteria

Isolation of probiotic LAB was carried out from the curd sample available in the local market. Two grams of sample were transferred in a flask consisting of de Man, Rogosa, and Sharpe (MRS) Broth (Hi-Media^®^, Mumbai, India) (100 mL) as enrichment media and incubated at 37 °C for 24 h. After the incubation period, 100 μL of the enriched sample was spread on MRS agar plates with incubation in anaerobic conditions for 48 h at 37 °C. Subsequently, purified bacterial colonies were sub-cultured. The purified bacterial colonies were maintained on a MRS agar medium for immediate use and stored at −20 °C in 20% glycerol for future use.

#### Identification of Lactic Acid Bacteria

Identification of the isolated lactic acid bacterial strain was carried out via the 16S rRNA gene sequencing method. Genomic DNA was extracted using a bacterial genomic DNA kit (GenElute^TM^, Sigma-Aldrich, Banglore, India). Quantification was carried out according to the method described by Sambrook et al. (1982) [75]. Optical density (OD) was measured (UV-1800, Shimadzu Spectrophotometer, Tokyo, Japan) at 260 and 290 nm. Furthermore, 0.8% agarose gel was used to confirm the purity of the extracted genomic DNA by electrophoresis. 16S rRNA gene amplification was carried out by using a pair of universal primers 27f (5′AGAGTTTGATCMTGGCTCAG3′) and 1492r (5′CGGTTACCTTGTTACGACTT3′). Final volume of 20 µL was used for the PCR amplification containing 10 pmol of each primer, 1x ReadyMix™ Taq PCR reaction mix (Sigma-Aldrich^®^, Bangalore, India), ~50 ng of template DNA, and to make up the total volume, nuclease free water was added. PCR cycling conditions were: 95 °C for 4 min, 35 cycles of 95 °C for 30 s, 54 °C for 30 s, and 72 °C for 1 min, followed by a final extension step at 72 °C for 5 min with a hold at −4 °C for ∞ time in a Thermal cycler (Applied Biosystems Veriti^®^, Lenexa, KS, USA). A 1% agarose gel was used to detect the amplified PCR products by electrophoresis. Staining was performed by ethidium bromide (EtBr), followed by visualization under UV light. Further purification of amplified PCR product was conducted using a GenElute™ PCR Clean-up kit (Sigma-Aldrich^®^, Bangalore, India) and the purified PCR product was sequenced. Finally, the Basic Local Alignment Search Tool (BLAST) on NCBI was used to carry out the sequence match analysis and sequences were later submitted to GenBank.

### 4.2. Biosurfactant Assays

A total of four LAB strains were isolated from the sample. All four isolates were first screened for their qualitative biosurfactant production ability. Out of all isolates displayed, positive results were selected for further assessments.

#### 4.2.1. Emulsification Assay

The potentiality of biosurfactants to emulsify n-hexadecane was carried out via an emulsification test [76]. Equal volume of n-hexadecane and cell-free biosurfactant solution were mixed by vortexing for 2 min and left to stand for 24 h. Calculation of an emulsification index (% EI24) was carried out by using the following equation.

$$\%E24=\frac{\mathrm{Height}\;\mathrm{of}\;\mathrm{formed}\;\mathrm{emulsion}}{\mathrm{Total}\;\mathrm{height}\;\mathrm{of}\;\mathrm{the}\;\mathrm{solution}}\times 100$$

#### 4.2.2. Drop Collapse Assay

The method described by Plaza et al. (2006) was followed to perform the drop collapse test [77]. First, a drop (35 µL) of the cell-free biosurfactant solution was put onto parafilm for observation of drop spreading on the parafilm surface after 15 min. The collapsed drop was scored as a positive result, which indicated the presence of biosurfactants.

#### 4.2.3. Oil Spreading Assay

The method described by Joe et al. (2019) was followed to perform the oil spreading assay [78]. First, distilled water (50 mL) was added to the Petri plates, followed by vegetable oil (100 µL) on the surface of the water. Later, on the oil surface, 10 µL of the cell-free biosurfactant solution was placed. After 30 s, the surface of the oil was visualized for the development of a clear zone.

#### 4.2.4. Blue Agar Plate (BAP) Assay

The BAP assay was carried out for the detection of biosurfactants on minimal agar medium consisting of cetyl trimethyl ammonium bromide (C-TAB, 0.4 mg/mL), glucose (2%), and methylene blue dye (0.2 mg/mL). Wells of a 6-mm size were made in the plates with the help of a sterile cork borer and 20 µL of cell-free biosurfactant solution was added into the wells. Plates were left in a refrigerator for 30 min for the diffusion of spent broth and kept for incubation for 24 h at 37 °C. After the incubation period, the presence of a dark blue colored halo around the wells was observed in the plates.

#### 4.2.5. Surface Tension (ST) Measurements

The surface tension was measured with a tensiometer (K11, Kruss, Hamburg, Germany). Pure water was used as a standard before reading (72.50 mN/m).

#### 4.2.6. Control Solutions

In the screening assays, phosphate-buffered saline (PBS) was used as a negative control and sodium dosecyl sulfate (SDS) was used as a positive control.

### 4.3. Production of Biosurfactant and Extraction

The production of biosurfactant by the isolated LAB strain was carried out by growing them in MRS-Lac broth (cultivation medium for LAB, where glucose is substituted by lactose). For the production of crude biosurfactant, overnight grown culture of *P. pentosaceus* (1%) was inoculated in MRS-Lac (1000 mL) medium and incubated at 37 °C for 48 h without shaking. After the incubation period, culture medium was placed for centrifugation (10,000 rpm, 10 min, 10 °C) to harvest the cells. Next, demineralized water was used to wash the cells twice and further re-suspended in 100 mL of phosphate buffered saline (PBS) (pH–7.0). This solution was stirred gently at room temperature for 4 h to release the cell associated biosurfactant. After 4 h, centrifugation was carried out to remove bacteria and the supernatant was collected by filtering with a 0.22 µm filter. In the end, the filtered and sterilized supernatant was lyophilized, stored at −40 °C, and further resuspended in deionized water at 100 mg/mL. This solution of the crude biosurfactant was then used for the biosurfactant assay and biofilm eradication assay.

### 4.4. Assessment of Biomass and Biosurfactant Concentration

Growth of bacteria was investigated by taking the OD of broth at 600 nm. For the determination of biomass, a regular method for the measurement of cell dry weight was employed. A total of 10 mL of the bacterial sample was transferred into pre-weighed tubes and centrifuged at 10,000 rpm for 10 min. Finally, cell pellets were collected and oven dried for 24 h at 100 °C and dry weight was eventually estimated. Biosurfactant concentration was determined according to the procedure described above and the concentration of produced biosurfactant was represented in mg/mL.

### 4.5. Assessment of Physical Properties of the Biosurfactant

To investigate the %EI24 of the produced biosurfactant by *P. pentosaceus*, an equal volume of n-hexadecane and the biosurfactant solution was mixed by 2 min vortexing and left to stand for 24 h. Then, calculation of an emulsification index (% EI24) was carried out as described above. Besides n-hexadecane, %EI24 of the produced biosurfactant was also investigated against various substrates such as gasoline, diesel, kerosene, toluene, olive oil, and sunflower oil. Furthermore, using a surface tensiometer, values of CMC and ST reduction were evaluated. CMC is defined as the abrupt discontinuity in the surface tension plot versus the plot of biosurfactant concentration.

### 4.6. Assessment of Antibacterial Activity

All pathogenic bacterial strains—*B. subtilis* (MTCC 121), *P. aeruginosa* (MTCC 741), *S. aureus* (MTCC 96), and *E. coli* (MTCC 9537)—were obtained from the Microbial Type Culture Collection (MTCC), India and maintained on Muller–Hinton Agar (MHA). Antibacterial activity of the *P. pentosaceus* crude biosurfactant and standard SDS was carried out via the agar cup/well diffusion method. First, bacterial cultures were grown overnight at 37 °C in a fresh medium and total of 0.5 McFarland standard 10^8^ colony-forming units/mL (CFU/mL) was matched by culture turbidity adjustment using sterile saline solution. Bacterial suspension was evenly spread all over the plates and wells were made with a sterile cork borer. A total of 60 µL of the *P. pentosaceus* crude biosurfactant solution (100 mg/mL) and SDS (1% V/V) solution was then inoculated into each respective well and plates were incubated at 37 °C for 24 h. Antibacterial activity was noted in the form of the zone of inhibition. Chloramphenicol and sterile water were used as positive and negative control, respectively.

### 4.7. Minimum Inhibitory Concentration (MIC) and Minimum Bactericidal Concentration (MBC) Determination

MIC determination of *P. pentosaceus* crude biosurfactant and standard SDS was carried out in microtiter plates (96-well) against test bacterial strains, as reported previously [79]. The inoculums were prepared from 12 h MHB culture. *P. pentosaceus* crude biosurfactant was diluted to two-fold ranging from 100 to 1.56 mg/mL in MHB in a 96-well plate (100 µL each well). Similarly, standard SDS was also diluted (1 to 0.2%). A diluted culture of each test bacteria was added to the respective well to control the final concentration of 10^8^ CFU/mL, followed by incubation for 24 h at 37 °C. MIC was then recorded as the lowest concentration at which absolute inhibition of observable growth was occurred. Wells with media were used as a negative control, whereas wells without biosurfactant in media, but only inoculated bacteria were used as a positive control. MBC determination was carried out similarly to the MIC assay by spreading 5 µL of sample from the wells, which did not exhibit evident growth on MHA plates, followed by incubation for 24 h at 37 °C. MBC was recorded as the lowest concentration at which 99% of the inoculum was killed (i.e., three or fewer colonies) [80].

### 4.8. Preparation of Biofilm

The crystal violet (CV) method was followed to determine the biofilm forming ability of the test strains using 96-well polystyrene plates [81]. Briefly, log phase culture of each test strain collectively with MHB (200 µL) at an initial turbidity of 0.05 at 600 nm was incubated for 24 h at 37 °C without shaking. After the incubation period, planktonic cells were removed by washing thrice with PBS and air-dried. A sample of 0.1% CV was then used for staining the wells and kept for 20 min. By dissolving in 95% ethanol, an excess amount of dye was taken out and absorbance was measured at 570 nm.

### 4.9. Antibiofilm Assays

#### 4.9.1. Effect of *P. pentosaceus* Crude Biosurfactant on Established Biofilms

The method described by Lemos et al. (2018) was followed to assess the efficacy of the *P. pentosaceus* crude biosurfactant and standard SDS on the established biofilms. The 96-well microtiter plates were used to form the biofilms by test strains containing 1% glucose, MHB, and cells (10^7^ cells/mL) [82]. Plates were incubated for 24 h at 37 °C. After incubation, planktonic cells were delicately removed from the wells with further washing of the wells with saline thrice. After washing, the *P. pentosaceus* crude biosurfactant and standard SDS (MIC) (200 µL) was then added to the respective wells and plates were kept for another day (24 h) of incubation at 37 °C. Absorbance (492 nm) was measured at 0 and after 24 h. MHB medium without any surfactant and with the individual test strain was used as the biofilm growth control and the biofilm eradication percentage was calculated as:

[OD (control)—OD (test)/OD (control)] × 100

#### 4.9.2. Effect of *P. pentosaceus* Crude Biosurfactant on Adherence of Biofilms

The method described by Plyuta et al. (2013) was followed to determine the effect of the *P. pentosaceus* crude biosurfactant and standard SDS on the adherence of biofilm formation [83]. Bacterial cell culture (100 µL) of each test strain (10^8^ CFU/mL) of the *P. pentosaceus* crude biosurfactant and standard SDS (MIC) collectively in respective 96-well microtiter plates were incubated for 24 h at 37 °C. After incubation, planktonic cells were delicately removed from the wells with further washing of the wells with PBS (200 µL). After washing, adhered cells were stained with 0.1% CV with 30 min incubation at 37 °C to visualize the developed biofilms by test strains. Excess CV dye was washed off with PBS and plates were finally fixed using 95% ethanol (200 µL) and incubation for 15 min at 37 °C. Absorbance at 590 nm was then measured. Inhibition percentage was calculated as:

[(OD (control)—OD (test)/OD control)] × 100(1)

### 4.10. Microscopic Assessment

#### 4.10.1. Determining and Visualization of Antibiofilm Activity by Light Microscopy

The method described by Musthafa et al. (2010) was followed to investigate the formed biofilms by test strains using LM with slight modifications [84]. The 24-well microtiter plates consisting of 1 × 1 cm^2^ size cover slips were inoculated with 500 µL of test cultures (10^8^ CFU/mL). In the same well, 500 µL of the *P. pentosaceus* crude biosurfactant (final concentration = MIC) was added as a treatment. For the positive control, the same volume of chloramphenicol was used with test strains. For the negative control, the same volume of sterile water was used with the test strains. After the incubation period of 24 h at 37 °C, glass cover slips with formed biofilms were gently removed and washed with PBS. Staining was performed with 0.1% CV as described above, followed by washing and air-drying for 5 min. Cover slips stained with CV were then observed under LM with 40× magnification (Axioscope A1, ZEISS, Oberkochen, Germany).

#### 4.10.2. Determining and Visualization of Antibiofilm Activity by Scanning Electron Microscopy

All test strain biofilms were also analyzed by SEM (with and without *P. pentosaceus* crude biosurfactant as well as the respective controls as described above). First, biofilms were fixed on glass cover slips using 2.5% glutaraldehyde at 37 °C for 30 min. After fixing, cover slips were washed thrice with PBS and then dehydrated through a graded series of ethanol solution (30%, 50%, 70%, 90%, and 100%) at 15 min interval. Samples were then freeze dried after the reinstation of ethanol with isoamyl acetate. Finally, using E-1010 ion sputter (Hitachi^®^, Tokyo, Japan), cover slips were coated with gold and observed under SEM (S-34002N SEM, Hitachi^®^, Tokyo, Japan).

### 4.11. Bacterial Metabolic Activity in Biofilms Assay

To determine the viability of bacterial cells within the biofilms, a colorimetric XTT reduction test was performed [65,85,86]. Briefly, the log phase culture of each test strain collectively in MHB (200 µL) with and without the *P. pentosaceus* crude biosurfactant at an initial turbidity of 0.1 at 600 nm was incubated for 24 h at 37 °C without shaking. After the incubation period, planktonic cells were removed by washing thrice with distilled water, followed by sterile PBS (100 µL). After washing, XTT-menadione (100 µL) solution (freshly prepared) was added into the wells. Plates were then incubated in the dark at 37 °C for 5 h. Following to the incubation, the colored supernatant (100 µL) was transferred from each well to a new 96-well microtiter plate. A microplate reader was then used to record the absorbance at 480 nm. Survival percentage of the bacterial population was determined as follows:

[(OD (biosurfactant treated sample)—OD (negative control)/OD of untreated control)] × 100

### 4.12. Bacterial Cell Damage Assay

To determine the damage to bacterial cells within the biofilms, the LDH assay was carried out. Briefly, the log phase culture of each test strain (100 µL) with MHB (100 µL) was added into 96-well microtiter plates and incubated for 24 h at 37 °C in static condition. After the incubation period, planktonic cells were discarded by washing thrice with sterile PBS (100 µL). *P. pentosaceus* crude biosurfactant (MIC) (100 µL) was then added and plates were kept for further incubation at 37 °C for 24 h in static condition. After incubation, LDH activity was then determined by collecting the supernatant using a LDH assay kit (Sigma Aldrich^®^, Bangalore, India) at 480 nm. Bacterial culture and MHB was used as a negative control.

### 4.13. Determining the Production of Exopolysaccharide by Ruthenium Red Staining

To determine the activity of the *P. pentosaceus* crude biosurfactant in diminishing the EPS matrix production of biofilm, a Ruthenium red staining assay was carried out [87]. Log phase culture of each test strain (100 µL) and the *P. pentosaceus* crude biosurfactant (MIC) were incubated for 24 h at 37 °C. After the incubation period, planktonic cells were discarded by washing thrice with sterile PBS (200 µL). Formed and structured biofilms by the adherent cells were then stained with 0.01% Ruthenium red (Sigma Aldrich^®^, Bangalore, India) (200 µL). One well without biofilm and with Ruthenium red, served as the blank. Plates were then kept for incubation at 37 °C for 1 h. Liquid holding the residual stain was resettled in a new microtiter plate and the absorbance was read at 450 nm. The quantity of the dye fixed to the biofilm matrix was measured as follows:

Abs_BF_ = Abs_B_ − Abs_S_

Whereas,

Abs_B_ = absorbance of blanks; and Abs_S_ = absorbance of residual stain collected from sample wells.

### 4.14. Assessment of Anti-Quorum Sensing (QS) Activity

To evaluate the anti-QS activity of the *P. pentosaceus* crude biosurfactant, well-diffusion assays using *C. violaceum* (MTCC 2656) was performed. An overnight culture of *C. violaceum* (100 µL) was spread over the LB plates and wells were punctured with the help of a gel puncture. A total of 60 µL of crude biosurfactant (100 mg/mL) was inoculated into the respective well and plates were incubated at 37 °C for 24 h. On the next day, zone of clearance was determined by confirming bacterial growth around the zone of clearance showing an anti-QS effect [70].

### 4.15. Quantification of Violacein

The inhibition of violacein in *C. violaceum* was determined as previously described by Blosser and Gray (2000) [88]. The overnight grown culture of *C. violaceum* (60 µL) was inoculated in sterile Erlenmeyer flasks with LB broth and incubated in the presence and absence of the *P. pentosaceus* crude biosurfactant. The flasks were incubated at 30 °C for 24 h. For quantification, bacterial cells were collected and dissolved in 1 mL DMSO. Then, cell debris was separated via centrifugation and the absorbance of soluble violacein was read at 585 nm. Percentage of violacein inhibition was determined as follows:

[(OD of control—OD of treated)/(OD of control)] × 100

### 4.16. Characterization of Crude Biosurfactant via Gas Chromatography-Mass Spectrophotometry (GC-MS) Analysis

The structural characterization of crude biosurfactants was performed via a Shimadzu Nexis GC-2030 Gas Chromatograph system equipped with a QP2020 NX Mass Spectrometer. The separation conditions were as follows: the starting temperature of the column was 100 °C for 1 min, then ramped at 30 °C to 270 °C, before a final hold at 270 °C for 10 min. A total of 10 µL of the sample was injected into the system and helium was used as a carrier gas. At the end of GC-MS separation, the obtained peaks were compared with the National Institute of Standards and Technology (NIST) database to determine the probable composition of the biosurfactants.

### 4.17. Statistical Analysis

All data were expressed as the mean ± standard deviation (SD) in triplicate. A significance test was carried out among the treatments by one way ANOVA followed by Tukey’s post hoc test at *p* < 0.05, except for the results of the antibiofilm activity. The values for the antibiofilm activities of the crude biosurfactant and standard SDS against selected pathogenic bacteria were assessed by unpaired *t*-tests at *p* < 0.05. All of the statistical analysis was conducted with software GraphPad Prism 5.0.

## 5. Conclusions

Overall, in the present study, we focused on production ability, characterizing a LAB-derived biosurfactant. Furthermore, we also demonstrated the antibacterial, anti-adhesive, and anti-biofilm potential of the crude biosurfactant extracted from *P. pentosaceus* against different biofilm-forming pathogenic bacteria. The isolated lactic acid producing probiotic strain *P. pentosaceus* has not been previously reported for the production of biosurfactants and the functional properties of the extracted crude biosurfactant are the prime findings of this study. Moreover, the results obtained advocated not only the prospective use of the *P. pentosaceus* crude biosurfactant as a green alternative antibacterial, antiadhesive, and antibiofilm agent, but also as a promising candidate to be employed in plenty of other areas such as the pharmaceutical and food industries. Despite the numerous advantages of biosurfactants, they are commercially unsuccessful compared to their synthetic counterparts due to high production cost and low yields. In future, the optimization of the *P. pentosaceus* biosurfactant production process should also be studied to achieve high yield and low extraction costs, which could bring novel biosurfactants to the commercial market. Further studies are also warranted to purify and individually test each GC-MS identified bioactive compound as well as to understand the overall mechanism, safety, and toxicity of the *P. pentosaceus* crude biosurfactant.

## Figures and Tables

**Table 1:** Qualitative and quantitative results of the different screening assays for the production of a cell-free biosurfactant (values are representatives of mean ± S.D (*n* = 3)).

Strain	Colony Characteristics	Gram’s Reaction	Oil Spreading Test	Drop Collapse Test	BAP Test	%E24 (n-Hexadecane)	ST (mN/m)
*P. pentosaceus*-MBP003	milky white, circular, concave, mucoid	Gram-positive cocci	Positive	Positive	Positive	39.73 ± 1.59	43.15 ± 1.17

**Table 2:** Antibacterial activity of the *P. pentosaceus* crude biosurfactant and standard SDS surfactant.

Bacterial Strain	*P. pentosaceus* Biosurfactant (mg/mL)		SDS (%)	
	MIC	MBC	MIC	MBC
*B. subtilis*	6.25	12.5	0.2	0.4
*E. coli*	12.5	25	0.4	0.6
*P. aeruginosa*	12.5	25	0.6	0.8
*S. aureus*	25	50	0.8	1

**Table 3:** Major constituents of the crude biosurfactant of *P. pentosaceus* using GC-MS.

No.	RT	% Area	Compound Name	Class
1	1.557	17.84	Glyceraldehyde	Sugar
2	1.752	3.09	Propanoic acid, 2-methyl-	Fatty Acyl
3	2.011	5.74	Butanoic acid, 2-methyl-	Fatty Acyl
4	2.474	16.91	Pentanoic acid, 4-methyl-	Fatty Acyl
5	3.417	0.17	Benzeneacetaldehyde	Aldehyde
6	4.082	0.88	Phenylethyl Alcohol	Alcohol
7	11.143	0.86	3-Methyl-2,3,6,7,8,8a-hexahydropyrrolo[1,2-alpyrazine-1,4-dione] (Cyclo(-d-Ala-L-Pro))	Diketopiperazine (Dipeptide)
8	11.627	1.14	Pyrrolo[1,2-a]pyrazine-1,4-dione, hexahydro-3-(1-methylethyl)- (Cyclo(-Pro-Val))	Diketopiperazine (Dipeptide)
9	13.683	1.39	n-Hexadecanoic acid	Fatty Acid (Surfactant)
10	15.382	16.70	Oleic Acid	Fatty Acid (Surfactant)
11	16.408	1.10	Octahydro-5H, 10H-dipyrrolo [1,2-a:1′,2′-d]pyrazine-5,10-dione	Diketopiperazine (Dipeptide)

## Data Availability

All data generated or analyzed during this study are included in this article.

## Supplementary Materials

The following are available online at https://www.mdpi.com/article/10.3390/antibiotics10111371/s1, Figure S1: Screening of biosurfactant production.

## References

[1] Patel M., Ashraf M.S., Siddiqui A.J., Ashraf S.A., Sachidanandan M., Snoussi M., Adnan M., Hadi S. (2020). Profiling and Role of Bioactive Molecules from *Puntius sophore* (Freshwater/Brackish Fish) Skin Mucus with Its Potent Antibacterial, Antiadhesion, and Antibiofilm Activities. Biomolecules.

[2] Adnan M., Patel M., Deshpande S., Alreshidi M., Siddiqui A.J., Reddy M.N., Emira N., De Feo V. (2020). Effect of *Adiantum philippense* Extract on Biofilm Formation, Adhesion with Its Antibacterial Activities Against Foodborne Pathogens, and Characterization of Bioactive Metabolites: An in vitro-in silico Approach. Front. Microbiol..

[3] Simoes M., Vieira M.J. Persister cells in Pseudomonas fluorescens biofilms treated with a biocide. Proceedings of the International Conference Processes in Biofilms: Fundamentals Applications.

[4] Sharma D., Sharma P.K., Malik A. (2011). Prevalence and antimicrobial susceptibility of drug resistant Staphylococcus aureus in raw milk of dairy cattle. Int. Res. J. Microbiol..

[5] Sharma D., Malik A. (2012). Incidence and prevalence of antimicrobial resistant *Vibrio cholerae* from dairy farms. Afr. J. Microbiol. Res..

[6] Sharma D., Saharan B.S., Chauhan N., Bansal A., Procha S. (2014). Production and structural characterization of *Lactobacillus helveticus* derived biosurfactant. Sci. World J..

[7] Sharma D., Saharan B.S., Chauhan N., Procha S., Lal S. (2015). Isolation and functional characterization of novel biosurfactant produced by Enterococcus faecium. SpringerPlus.

[8] Sharma D., Saharan B.S. (2016). Functional characterization of biomedical potential of biosurfactant produced by Lactobacillus helveticus. Biotechnol. Rep..

[9] Saharan B.S., Sharma D., Sahu R., Sahin O., Warren A. (2013). Towards algal biofuel production: A concept of green bio energy development. Innov. Rom. Food Biotechnol..

[10] Van Hamme J.D., Singh A., Ward O.P. (2006). Physiological aspects: Part 1 in a series of papers devoted to surfactants in microbiology and biotechnology. Biotechnol. Adv..

[11] Cameotra S.S., Makkar R.S., Kaur J., Mehta S.K. (2010). Synthesis of biosurfactants and their advantages to microorganisms and mankind. Biosurfactants.

[12] Singh P., Cameotra S.S. (2004). Potential applications of microbial surfactants in biomedical sciences. TRENDS Biotechnol..

[13] Sharma D., Singh Saharan B. (2014). Simultaneous production of biosurfactants and bacteriocins by probiotic *Lactobacillus casei* MRTL3. Int. J. Microbiol..

[14] Thavasi R., Jayalakshmi S., Banat I.M. (2011). Effect of biosurfactant and fertilizer on biodegradation of crude oil by marine isolates of *Bacillus megaterium*, *Corynebacterium kutscheri* and *Pseudomonas aeruginosa*. Bioresour. Technol..

[15] Gudiña E.J., Teixeira J.A., Rodrigues L.R. (2011). Biosurfactant-producing lactobacilli: Screening, production profiles, and effect of medium composition. Appl. Environ. Soil Sci..

[16] Saravanakumari P., Mani K. (2010). Structural characterization of a novel xylolipid biosurfactant from Lactococcus lactis and analysis of antibacterial activity against multi-drug resistant pathogens. Bioresour. Technol..

[17] Falagas M.E., Makris G.C. (2009). Probiotic bacteria and biosurfactants for nosocomial infection control: A hypothesis. J. Hosp. Infect..

[18] Rivera O.M.P., Moldes A.B., Torrado A.M., Domínguez J.M. (2007). Lactic acid and biosurfactants production from hydrolyzed distilled grape marc. Process Biochem..

[19] Rodrigues L., Banat I.M., Teixeira J., Oliveira R. (2006). Biosurfactants: Potential applications in medicine. J. Antimicrob. Chemother..

[20] Rodrigues L., Van der Mei H.C., Teixeira J., Oliveira R. (2004). Influence of biosurfactants from probiotic bacteria on formation of biofilms on voice prostheses. Appl. Environ. Microbiol..

[21] Servin A.L. (2004). Antagonistic activities of lactobacilli and bifidobacteria against microbial pathogens. FEMS Microbiol. Rev..

[22] Heinemann C., van Hylckama Vlieg J.E., Janssen D.B., Busscher H.J., van der Mei H.C., Reid G. (2000). Purification and characterization of a surface-binding protein from Lactobacillus fermentum RC-14 that inhibits adhesion of Enterococcus faecalis 1131. FEMS Microbiol. Lett..

[23] Benincasa M., Abalos A., Oliveira I., Manresa A. (2004). Chemical structure, surface properties and biological activities of the biosurfactant produced by Pseudomonas aeruginosa LBI from soapstock. Antonie Van Leeuwenhoek.

[24] Velraeds M., Van der Mei H., Reid G., Busscher H.J. (1996). Inhibition of initial adhesion of uropathogenic *Enterococcus faecalis* by biosurfactants from *Lactobacillus isolates*. Appl. Environ. Microbiol..

[25] Walencka E., Różalska S., Sadowska B., Różalska B. (2008). The influence of *Lactobacillus acidophilus*-derived surfactants on staphylococcal adhesion and biofilm formation. Folia Microbiol..

[26] Rodrigues L.R., Teixeira J.A., van der Mei H.C., Oliveira R. (2006). Isolation and partial characterization of a biosurfactant produced by Streptococcus thermophilus A. Colloids Surf. B Biointerfaces.

[27] Rodrigues L.R., Teixeira J.A., van der Mei H.C., Oliveira R. (2006). Physicochemical and functional characterization of a biosurfactant produced by Lactococcus lactis 53. Colloids Surf. B Biointerfaces.

[28] Rodrigues L., Van der Mei H., Teixeira J.A., Oliveira R. (2004). Biosurfactant from *Lactococcus lactis* 53 inhibits microbial adhesion on silicone rubber. Appl. Microbiol. Biotechnol..

[29] Busscher H.J., Van Hoogmoed C., Geertsema-Doornbusch G.I., Van der Kuijl-Booij M., Van der Mei H. (1997). Streptococcus thermophilus and its biosurfactants inhibit adhesion by *Candida* spp. on silicone rubber. Appl. Environ. Microbiol..

[30] Gan B.S., Kim J., Reid G., Cadieux P., Howard J.C. (2002). Lactobacillus fermentum RC-14 inhibits *Staphylococcus aureus* infection of surgical implants in rats. J. Infect. Dis..

[31] Singh A., Van Hamme J.D., Ward O.P. (2007). Surfactants in microbiology and biotechnology: Part 2. Application aspects. Biotechnol. Adv..

[32] Adnan M., Morton G., Singh J., Hadi S. (2010). Contribution of rpoS and bolA genes in biofilm formation in *Escherichia coli* K-12 MG1655. Mol. Cell. Biochem..

[33] Song Y.J., Yu H.H., Kim Y.J., Lee N.-K., Paik H.-D. (2019). Anti-biofilm activity of grapefruit seed extract against *Staphylococcus aureus* and *Escherichia coli*. J. Microbiol. Biotechnol..

[34] Adnan M., Alshammari E., Patel M., Ashraf S.A., Khan S., Hadi S. (2018). Significance and potential of marine microbial natural bioactive compounds against biofilms/biofouling: Necessity for green chemistry. PeerJ.

[35] Patel K., Patel M. (2020). Improving bioremediation process of petroleum wastewater using biosurfactants producing *Stenotrophomonas* sp. S1VKR-26 and assessment of phytotoxicity. Bioresour. Technol..

[36] Adnan M., Alshammari E., Ashraf S.A., Patel K., Lad K., Patel M. (2018). Physiological and molecular characterization of biosurfactant producing endophytic fungi *Xylaria regalis* from the cones of *Thuja plicata* as a potent plant growth promoter with its potential application. BioMed. Res. Int..

[37] Kumar A., Singh S.K., Kant C., Verma H., Kumar D., Singh P.P., Modi A., Droby S., Kesawat M.S., Alavilli H. (2021). Microbial Biosurfactant: A New Frontier for Sustainable Agriculture and Pharmaceutical Industries. Antioxidants.

[38] Dusane D., Rajput J., Kumar A., Nancharaiah Y., Venugopalan V., Zinjarde S. (2008). Disruption of fungal and bacterial biofilms by lauroyl glucose. Lett. Appl. Microbiol..

[39] Dusane D.H., Nancharaiah Y.V., Zinjarde S.S., Venugopalan V.P. (2010). Rhamnolipid mediated disruption of marine *Bacillus pumilus* biofilms. Colloids Surf. B Biointerfaces.

[40] Rivardo F., Turner R.J., Allegrone G., Ceri H., Martinotti M. (2009). Anti-adhesion activity of two biosurfactants produced by *Bacillus* spp. prevents biofilm formation of human bacterial pathogens. Appl. Microbiol. Biotechnol..

[41] Amaral P., Da Silva J., Lehocky M., Barros-Timmons A., Coelho M., Marrucho I., Coutinho J. (2006). Production and characterization of a bioemulsifier from *Yarrowia lipolytica*. Process Biochem..

[42] de Jesús Cortés-Sánchez A., Hernández-Sánchez H., Jaramillo-Flores M.E. (2013). Biological activity of glycolipids produced by microorganisms: New trends and possible therapeutic alternatives. Microbiol. Res..

[43] Mulligan C.N., Gibbs B.F. (2004). Types, production and applications of biosurfactants. Proc. Indian Natl. Sci. Acad. USA Part B.

[44] Mulligan C.N. (2005). Environmental applications for biosurfactants. Environ. Pollut..

[45] Pinto S., Alves P., Santos A., Matos C., Oliveiros B., Gonçalves S., Gudiña E., Rodrigues L., Teixeira J., Gil M. (2011). Poly (dimethyl siloxane) surface modification with biosurfactants isolated from probiotic strains. J. Biomed. Mater. Res. Part A.

[46] Hua Z., Chen J., Lun S., Wang X. (2003). Influence of biosurfactants produced by Candida antarctica on surface properties of microorganism and biodegradation of n-alkanes. Water Res..

[47] Satpute S., Bhawsar B., Dhakephalkar P., Chopade B. (2008). Assessment of different screening methods for selecting biosurfactant producing marine bacteria. NISCAIR Online Period. Repos..

[48] Thavasi R., Jayalakshmi S., Balasubramanian T., Banat I.M. (2008). Production and characterization of a glycolipid biosurfactant from *Bacillus megaterium* using economically cheaper sources. World J. Microbiol. Biotechnol..

[49] Desai J.D., Banat I.M. (1997). Microbial production of surfactants and their commercial potential. Microbiol. Mol. Biol. Rev..

[50] Abalos A., Pinazo A., Infante M., Casals M., Garcia F., Manresa A. (2001). Physicochemical and antimicrobial properties of new rhamnolipids produced by Pseudomonas a eruginosa AT10 from soybean oil refinery wastes. Langmuir.

[51] Arutchelvi J.I., Bhaduri S., Uppara P.V., Doble M. (2008). Mannosylerythritol lipids: A review. J. Ind. Microbiol. Biotechnol..

[52] Sánchez M., Aranda F.J., Teruel J.A., Ortiz A. (2011). New pH-sensitive liposomes containing phosphatidylethanolamine and a bacterial dirhamnolipid. Chem. Phys. Lipids.

[53] Neu T.R. (1996). Significance of bacterial surface-active compounds in interaction of bacteria with interfaces. Microbiol. Rev..

[54] Tahmourespour A., Salehi R., Kermanshahi R.K., Eslami G. (2011). The anti-biofouling effect of *Lactobacillus fermentum*-derived biosurfactant against *Streptococcus mutans*. Biofouling.

[55] Pornsunthorntawee O., Wongpanit P., Chavadej S., Abe M., Rujiravanit R. (2008). Structural and physicochemical characterization of crude biosurfactant produced by Pseudomonas aeruginosa SP4 isolated from petroleum-contaminated soil. Bioresour. Technol..

[56] Vaz D.A., Gudiña E.J., Alameda E.J., Teixeira J.A., Rodrigues L.R. (2012). Performance of a biosurfactant produced by a *Bacillus subtilis* strain isolated from crude oil samples as compared to commercial chemical surfactants. Colloids Surf. B Biointerfaces.

[57] Abdalsadiq N., Hassan Z., Lani M. (2018). Antimicrobial, antiadhesion and anti-biofilm activity of biosurfactants isolated from *lactobacillus* spp.. Life Sci. Inf. Publ..

[58] Schlusselhuber M., Godard J., Sebban M., Bernay B., Garon D., Seguin V., Oulyadi H., Desmasures N. (2018). Characterization of Milkisin, a Novel Lipopeptide with Antimicrobial Properties Produced by *Pseudomonas* sp. UCMA 17988 Isolated from Bovine Raw Milk. Front. Microbiol..

[59] Banat I.M., De Rienzo M.A., Quinn G.A. (2014). Microbial biofilms: Biosurfactants as antibiofilm agents. Appl. Microbiol. Biotechnol..

[60] Paes Leme A.F., Koo H., Bellato C.M., Bedi G., Cury J.A. (2006). The role of sucrose in cariogenic dental biofilm formation—New insight. J. Dent. Res..

[61] Kim D., Hwang G., Liu Y., Wang Y., Singh A.P., Vorsa N., Koo H. (2015). Cranberry Flavonoids Modulate Cariogenic Properties of Mixed-Species Biofilm through Exopolysaccharides-Matrix Disruption. PLoS ONE.

[62] Lentino J.R. (2003). Prosthetic joint infections: Bane of orthopedists, challenge for infectious disease specialists. Clin. Infect. Dis..

[63] Parkinson M. (1985). Bio-surfactants. Biotechnol. Adv..

[64] Van Hoogmoed C.G., van Der Kuijl-Booij M., van Der Mei H.C., Busscher H.J. (2000). Inhibition of Streptococcus mutans NS adhesion to glass with and without a salivary conditioning film by biosurfactant-releasing Streptococcus mitis strains. Appl. Environ. Microbiol..

[65] Ramage G., Vande Walle K., Wickes B.L., López-Ribot J.L. (2001). Standardized method for in vitro antifungal susceptibility testing of *Candida albicans* biofilms. Antimicrob. Agents Chemother..

[66] Augustine N., Kumar P., Thomas S. (2010). Inhibition of Vibrio cholerae biofilm by AiiA enzyme produced from *Bacillus* spp.. Arch. Microbiol..

[67] Gudiña E.J., Rocha V., Teixeira J.A., Rodrigues L.R. (2010). Antimicrobial and antiadhesive properties of a biosurfactant isolated from *Lactobacillus paracasei* ssp. paracasei A20. Lett. Appl. Microbiol..

[68] Fracchia L.M., Cavallo G., Allegrone M.G., Martinotti A. (2010). *Lactobacillus*-derived biosurfactant inhibits biofilm formation of human pathogenic *Candida albicans* biofilm producers. Appl. Microbiol. Microb. Biotechnol..

[69] Van der Mei H.C., Free R.H., Elving G.J., Van Weissenbruch R., Albers F.W.J., Busscher H.J. (2000). Effect of probiotic bacteria on prevalence of yeasts in oropharyngeal biofilms on silicone rubber voice prostheses in vitro. J. Med. Microbiol..

[70] McLean R.J.C., Pierson L.S., Fuqua C. (2004). A simple screening protocol for the identification of quorum signal antagonists. J. Microbiol. Methods.

[71] Packiavathy I.A.S.V., Agilandeswari P., Musthafa K.S., Pandian S.K., Ravi A.V. (2012). Antibiofilm and quorum sensing inhibitory potential of *Cuminum cyminum* and its secondary metabolite methyl eugenol against Gram negative bacterial pathogens. Food Res. Int..

[72] Husain F.M., Ahmad I., Asif M., Tahseen Q. (2013). Influence of clove oil on certain quorum-sensing-regulated functions and biofilm of *Pseudomonas aeruginosa* and *Aeromonas hydrophila*. J. Biosci..

[73] Ghasemi A., Moosavi-Nasab M., Setoodeh P., Mesbahi G., Yousefi G. (2019). Biosurfactant production by lactic acid bacterium Pediococcus dextrinicus SHU1593 grown on different carbon sources: Strain screening followed by product characterization. Sci. Rep..

[74] Velraeds M.M., van der Mei H.C., Reid G., Busscher H.J. (1996). Physicochemical and biochemical characterization of biosurfactants released by *Lactobacillus* strains. Colloids Surf. B Biointerfaces.

[75] Sambrook J.F.E., Maniatis T. (1982). Molecular Cloning: A Laboratory Manual.

[76] Satpute S.K., Banpurkar A.G., Dhakephalkar P.K., Banat I.M., Chopade B.A. (2010). Methods for investigating biosurfactants and bioemulsifiers: A review. Crit. Rev. Biotechnol..

[77] Płaza G.A., Zjawiony I., Banat I.M. (2006). Use of different methods for detection of thermophilic biosurfactant-producing bacteria from hydrocarbon-contaminated and bioremediated soils. J. Pet. Sci. Eng..

[78] Joe M.M., Gomathi R., Benson A., Shalini D., Rengasamy P., Henry A.J., Truu J., Truu M., Sa T. (2019). Simultaneous Application of Biosurfactant and Bioaugmentation with Rhamnolipid-Producing Shewanella for Enhanced Bioremediation of Oil-Polluted Soil. Appl. Sci..

[79] Wiegand I., Hilpert K., Hancock R.E. (2008). Agar and broth dilution methods to determine the minimal inhibitory concentration (MIC) of antimicrobial substances. Nat. Protoc..

[80] Pereira Silveira C., Torres-Rodríguez J.M., Alvarado-Ramírez E., Murciano-Gonzalo F., Dolande M., Panizo M., Reviakina V. (2009). MICs and minimum fungicidal concentrations of amphotericin B, itraconazole, posaconazole and terbinafine in Sporothrix schenckii. J. Med. Microbiol..

[81] Lee K.W., Periasamy S., Mukherjee M., Xie C., Kjelleberg S., Rice S.A. (2014). Biofilm development and enhanced stress resistance of a model, mixed-species community biofilm. ISME J..

[82] Lemos A.S.O., Campos L.M., Melo L., Guedes M., Oliveira L.G., Silva T.P., Melo R.C.N., Rocha V.N., Aguiar J.A.K., Apolônio A.C.M. (2018). Antibacterial and Antibiofilm Activities of Psychorubrin, a Pyranonaphthoquinone Isolated From *Mitracarpus frigidus* (Rubiaceae). Front. Microbiol..

[83] Plyuta V., Zaitseva J., Lobakova E., Zagoskina N., Kuznetsov A., Khmel I. (2013). Effect of plant phenolic compounds on biofilm formation by Pseudomonas aeruginosa. APMIS.

[84] Musthafa K.S., Ravi A.V., Annapoorani A., Packiavathy I.S., Pandian S.K. (2010). Evaluation of anti-quorum-sensing activity of edible plants and fruits through inhibition of the N-acyl-homoserine lactone system in *Chromobacterium violaceum* and *Pseudomonas aeruginosa*. Chemotherapy.

[85] Nett J.E., Cain M.T., Crawford K., Andes D.R. (2011). Optimizing a *Candida biofilm* microtiter plate model for measurement of antifungal susceptibility by tetrazolium salt assay. J. Clin. Microbiol..

[86] Siddiqui A.J., Bhardwaj J., Goyal M., Prakash K., Adnan M., Alreshidi M.M., Patel M., Soni A., Redman W. (2020). Immune responses in liver and spleen against Plasmodium yoelii pre-erythrocytic stages in Swiss mice model. J. Adv. Res..

[87] Borucki M.K., Krug M.J., Muraoka W.T., Call D.R. (2003). Discrimination among *Listeria monocytogenes* isolates using a mixed genome DNA microarray. Vet. Microbiol..

[88] Blosser R.S., Gray K.M. (2000). Extraction of violacein from *Chromobacterium violaceum* provides a new quantitative bioassay for N-acyl homoserine lactone autoinducers. J. Microbiol. Methods.

