# Supplementary material for: Functional and Structural Characterization of Pediococcus pentosaceus-Derived Biosurfactant and Its Biomedical Potential against Bacterial Adhesion, Quorum Sensing, and Biofilm Formation
Source: Antibiotics (Basel). 2021 Nov 9;10(11):1371. doi: 10.3390/antibiotics10111371 (PMC8614858; doi:10.3390/antibiotics10111371)
Supplement: Supplementary file 1 [file antibiotics-10-01371-s001.zip › antibiotics-1455779-supplementary.pdf]

antibiotics

MDPI

Article

Functional and Structural Characterization of *Pediococcus pentosaceus*-Derived Biosurfactant and Its Biomedical Potential against Bacterial Adhesion, Quorum Sensing, and Biofilm Formation

Mohd Adnan [1,*], Arif Jamal Siddiqui [1], Walid Sabri Hamadou [1], Syed Amir Ashraf [2], Md Imtaiyaz Hassan [3], Mejdi Snoussi [1,4], Riadh Badraoui [1,5], Arshad Jamal [1], Fevzi Bardakci [1], Amir Mahgoub Awadelkareem [2], Manojkumar Sachidanandan [6] and Mitesh Patel [7,*]

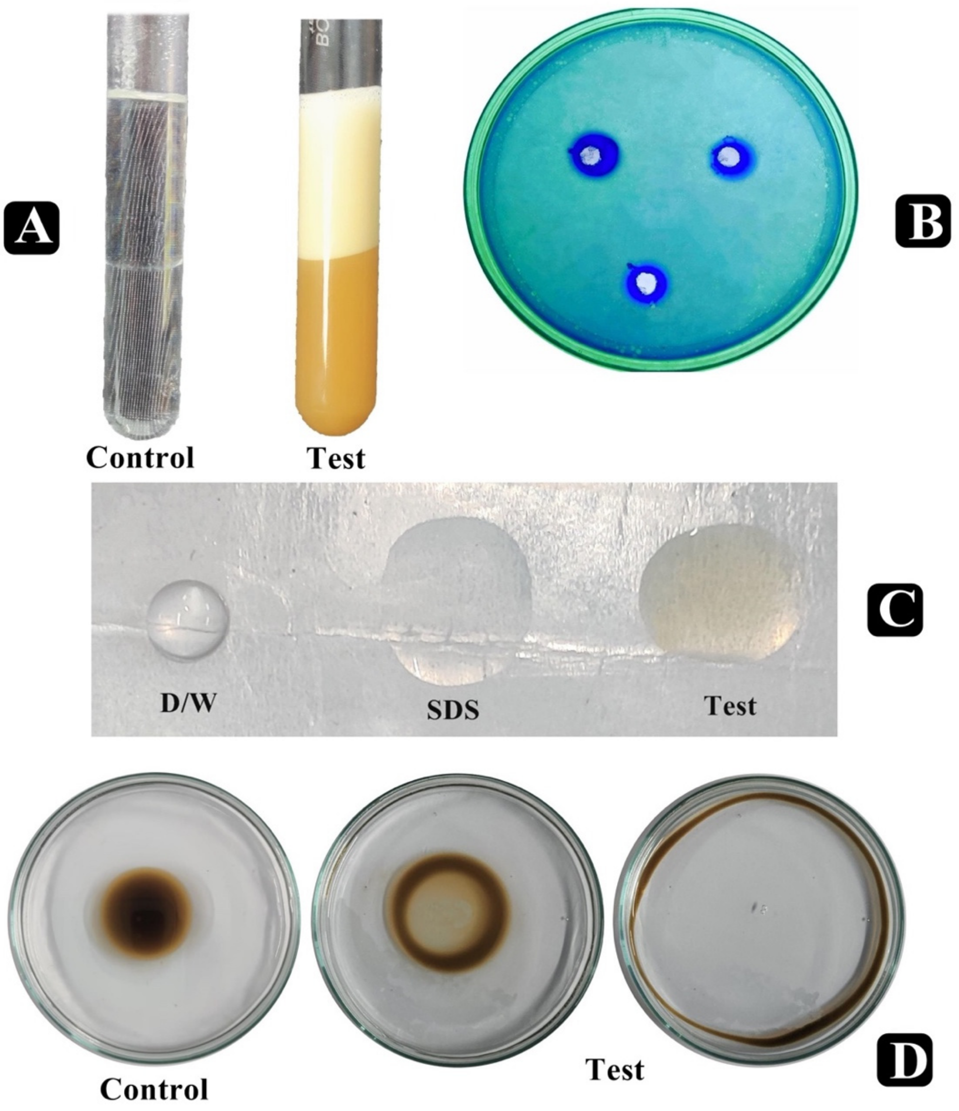

Figure S1. Screening of biosurfactant production A) Emulsification index test, B) Blue agar plate assay, C) Drop collapse assay, D) Oil spreading assay.
